# Love at First Sight

**DOI:** 10.3389/fpsyg.2016.01113

**Published:** 2016-07-28

**Authors:** James A. Grant-Jacob

**Affiliations:** Faculty of Physical Sciences and Engineering, University of SouthamptonSouthampton, UK

**Keywords:** interpersonal attraction, love, emotion, interpersonal relationships, perception

Evolved traits such as intuitive skills have allowed individuals to quickly evaluate a suitable sexual partner in about 100 ms (Olson and Marshuetz, 2005; Willis and Todorov, 2006; Todorov, 2008). Having determined that someone is attractive at first sight, an individual may become emotionally attached to that person and communicate their affection via a copulatory gaze, in which the individual gazes into the other person's eyes for many seconds (Fisher, 1992). In this opinion piece, I discuss why an individual can become attracted to and emotionally attached to another person in an instant at first sight and I propose how such feelings may be requited and thus how love at first sight between two people can occur if the receiver of such attraction has similar attributes to the individual.

A person's perceived attractiveness can vary depending on the individual who is perceiving their attractiveness. Studies on attraction have shown that people are strongly sexually attracted to lookalikes in physical appearance (Folkes, 1982; Alvarez and Bolívar, 2004; Malin, 2004), with researchers having found that subjects rated pictures of faces morphed with their own face as being more attractive (Penton-Voak et al., 1999). In work by Zaidel et al. (2003), subjects found that those faces that were more attractive, were also more trustworthy. An individual's personality and trustworthiness can be perceived in the subtle nonverbal behavioral information of such primary facial features as the eyes (Argyle, 1988; Little and Perrett, 2007) and we can perceive from someone's face whether they are happy, sad, well or ill (Rhodes and Zebrowitz, 2002; Ekman and Friesen, 2003). We can sometimes even perceive sexual orientation (Rule et al., 2009) and perhaps intelligence (Zebrowitz et al., 2002; Kanazawa, 2011). As such, I believe it is our ability to perceive another individual's personality that can allow us to be attracted to someone before we have even spoken to them.

Researchers have shown that interpersonal attraction is positively correlated to personality similarity, with positive people preferring to be with other positive people (Byrne and Griffitt, 1973), and individuals are also attracted to others who are similar in culture, economic status and socialness (Byrne et al., 1966; Berscheid et al., 1971; Buss and Barnes, 1986). As such, in my opinion, since an individual is generally attracted to someone who they believe they can trust and perhaps be in a happy relationship with, and usually, since that person is someone who has a personality similar to their own, those people who look similar to the individual are perceived as having a similar personality to that individual, and are therefore perceived to be more attractive. In addition, because individuals may inherit similar facial features to their parents, those who look like the individual may also be seen as trustworthy, since they may be a reminder of trusting faces from childhood (Perrett, 2010). Such belief in a lookalike being trustworthy and suitable relationship material is not unfounded in the literature, as couples who have similar physical attributes have shown to demonstrate strong relationship commitment (Murstein, 1972) and relationship stability (Kurdek and Schnopp-Wyatt, 1997). Although, the apparent superficial manner of falling in love at first sight could mean an individual may become attached to someone they have nothing in common with, and thus potentially be involved in a relationship that may not last long, the positive impact of the first impression can compensate the superficial manner of attraction at first sight (Sunnafrank and Ramirez, 2004; Barelds and Barelds-Dijkstra, 2007), and as discussed by Tops et al. (2014), the positive initial impact can become replaced with familiarity and predictability of the partner, which can lead to a potentially long-term attraction.

The attraction of the individual to another person can be conveyed by their eyes, i.e., via copulatory gaze, such that the more an individual likes that person, the longer they want to gaze into their eyes in order to express the strength of our feelings (Rubin, 1970). Therefore, after having looked into the other person's eyes to determine if they are attractive, the individual may continue to gaze into the other person's eyes for many seconds. By an individual conveying their feelings of attraction to the person they are attracted to, the receiver of such attraction may be attracted back to the individual, not only because they are being appraised as being a suitable partner, and as suggested by Myers (2012), there is essentially less energy needed for them to claim a mate, but in my view, because the individual may attracted to the receiver because the receiver is a look-a-like, the receiver may in turn perceive the individual as having similar attributes and thus perceived as having a suitable personality too. As such, the receiver may gaze back into the individuals' eyes to convey that they like them. The gazing back may reinforce the individual's belief that the receiver of their attraction is suitable, and as both individuals gaze into each other's eyes, they perhaps realize that they are both attracted to each other, and so an immediate unspoken emotional union is potentially formed in which both individuals become emotionally attached to each other, and love at first sight occurs between two people at the same time.

A comparative study of individuals in relationships formed from reciprocated love at first sight, which involves discussions of the individuals' initial attraction, as well as the individuals' perceived personalities before and during the relationship, will hopefully explore the ideas expressed in this work as well as aid in our understanding of the prevalence and mechanics of love at first sight.

## Author contributions

The author confirms being the sole contributor of this work and approved it for publication.

### Conflict of interest statement

The author declares that the research was conducted in the absence of any commercial or financial relationships that could be construed as a potential conflict of interest.
